# Proteomic and Functional Analysis Reveals Temperature-Driven Immune Evasion Strategies of *Streptococcus iniae* in Yellowfin Seabream (*Acanthopagrus latus*)

**DOI:** 10.3390/biology14080986

**Published:** 2025-08-02

**Authors:** Yanjian Yang, Guanrong Zhang, Ruilong Xu, Yiyang Deng, Zequan Mo, Yanwei Li, Xueming Dan

**Affiliations:** 1 Nansha-South China Agricultural University Fishery Research Institute, Guangzhou 511457, China; yangyanjian@gznshnyyyjy1.wecom.work (Y.Y.); zhang-gr@139.com (G.Z.); 2 University Joint Laboratory of Guangdong Province, Hong Kong and Macao Region on Marine Bioresource Conservation and Exploitation, Guangdong Laboratory for Lingnan Modern Agriculture, College of Marine Sciences, South China Agricultural University, Guangzhou 510642, China; xrl@stu.scau.edu.cn (R.X.); 20233140009@stu.scau.edu.cn (Y.D.); mzq1990@scau.edu.cn (Z.M.); yanweili@scau.edu.cn (Y.L.)

**Keywords:** *Streptococcus iniae*, temperature, virulence factors, immune evasion, yellowfin seabream, cell function

## Abstract

*Streptococcus iniae* (*S. iniae*) is a globally significant aquatic pathogen responsible for severe economic losses in aquaculture. While the *S. iniae* infection often exhibits distinct seasonal patterns strongly correlated with water temperature, there is limited knowledge regarding the temperature-dependent immune evasion strategies of *S. iniae*. Our results demonstrated a striking temperature-dependent virulence phenotype, with significantly higher *A. latus* mortality rates observed at high temperature (HT, 33 °C) compared to low temperature (LT, 23 °C). Proteomic analysis revealed temperature-dependent upregulation of key virulence factors, including streptolysin S-related proteins (SagG, SagH), antioxidant-related proteins (SodA), and multiple capsular polysaccharide (cps) synthesis proteins (cpsD, cpsH, cpsL, cpsY). Flow cytometry analysis showed that HT infection significantly reduced the percentage of lymphocyte and myeloid cell populations in the head kidney leukocytes of *A. latus*, which was associated with elevated *caspase-3/7* expression and increased apoptosis. In addition, HT infection significantly inhibited the release of reactive oxygen species (ROS) but not nitric oxide (NO) production. Using *S. iniae* cps-deficient mutant, Δcps, we demonstrated that the cps is essential for temperature-dependent phagocytosis resistance in *S. iniae*, as phagocytic activity against Δcps remained unchanged across temperatures, while NS-1 showed significantly reduced uptake at HT. These findings provide new insights into the immune evasion of *S. iniae* under thermal regulation, deepening our understanding of the thermal adaptation of aquatic bacterial pathogens.

## Simple Summary

*Streptococcus iniae* (*S. iniae*) is a major aquaculture pathogen causing significant economic losses. While infections show seasonal patterns tied to water temperature, the mechanisms behind its thermal adaptation remain unclear. This study explored the proteomic changes in *S. iniae* at different temperatures and their effects on immune responses in yellowfin seabream (*Acanthopagrus latus*, *A. latus*). Results demonstrated that elevated temperatures markedly upregulated bacterial virulence factor expression, resulting in significantly higher host mortality rates. Furthermore, thermal stress enhances the destruction of key immune functions of the host by *S. iniae*, including immune cell survival, ROS production, and phagocytic activity. These findings provide novel insights into the heat-regulated immune evasion strategies of *S. iniae*, offering valuable theoretical foundations for developing temperature-aware disease management protocols in aquaculture systems.

## 1. Introduction

The yellowfin seabream (*Acanthopagrus latus*, *A. latus*), which can withstand a wide range of salinities and temperatures [1,2], is extensively distributed across the warm coastal areas of the Indo-West Pacific, including the coastlines of China, Korea, Japan, and Vietnam [3]. In China, *A. latus* is a national-level agricultural product with a geographical indication, which is of great significance for promoting local economic development [4]. In addition, *A. latus* plays an important ecological role and is regarded as an ideal species for marine cage culture. *Streptococcus iniae* (*S. iniae*) stands out as one of the most prevalent Gram-positive pathogens in *A. latus* aquaculture [5]. Owing to its high infectivity and the consequent high mortality rate among fish, it has garnered increasing attention and incurred significant economic losses in the *A. latus* farming sector.

*S. iniae* is an emerging zoonotic Gram-positive pathogen that was first isolated from a freshwater dolphin with skin lesions in 1976 [6,7]. In 1981, *S. iniae* was observed in farmed freshwater rainbow trout (*Oncorhynchus mykiss*), ayu sweetfish (*Plecoglossus altivelis*), and tilapia (*Oreochromis*) at various districts of Japan [8]. Since then, *S. iniae* infections have been detected in many species of saltwater and freshwater fish, including Japanese flounder (*Paralichthys olivaceus*) [9], red drum (*Sciaenops ocellatus*) [10], red porgy (*Pagrus pagrus, L.*) [11], channel catfish (*Ictalurus punctatus*) [12], Siberian sturgeon (*Acipenser baerii*) [13], hybrid tilapia (Nile tilapia *Oreochromis niloticus* × Mozambique tilapia *O. mossambicus*) [14], mandarin fish (*Siniperca chuatsi*) [15], Derbio (*Trachinotus ovatus*) [16], Adriatic sturgeon (*Acipenser naccarii*) [17], and yellow catfish (*Tachysurus fulvidraco*) [18]. In aquaculture, *S. iniae* can lead to invasive infections in fish following skin damage [19]. Fish infected with this bacterium typically exhibit symptoms such as abnormal swimming behavior, meningitis, and sepsis [20]. *S. iniae*-induced fish diseases can lead to high mortality and cause huge losses to the aquaculture industry [21].

The outbreak of *S. iniae* disease is a complex process influenced by multiple factors. Although the virulence of pathogenic bacteria is undoubtedly one of the key factors triggering this disease, environmental factors, such as temperature and pH value, also play a crucial role in the disease’s occurrence and development [22]. The association between the water environment temperature and *S. iniae* disease is particularly salient. In 2016, a massive die-off of wild marine fish caused by *S. iniae* was observed in Western Australia; the higher water temperature was considered a key factor in the outbreak [23]. In the epidemiological survey of Australian lungfish, it was also confirmed that there was a close correlation between the mortality rate caused by *S. iniae* and water temperature [24]. In addition, *S. iniae* exhibits accelerated growth and heightened virulence at a culture temperature of 35 °C, and transcriptome technology confirmed that temperature could regulate the transcription levels of *S. iniae* genes [25]. However, the expression of virulence proteins of *S. iniae* at high temperatures and its effect on the function of fish immune cells has not yet been described.

In this study, the effects of *S. iniae* on the survival of *A. latus* at 23 °C and 33 °C were investigated. Subsequently, the proteomic analysis was performed on *S. iniae* grown at these temperatures to identify differentially expressed proteins, followed by functional enrichment analysis. Based on the proteomic findings, we further examined the effects of *S. iniae* on the immune responses of *A. latus* head kidney lymphocytes and myeloid cells, including apoptosis level, nitric oxide (NO) and reactive oxygen species (ROS) production, and phagocytic activity. Notably, we discovered that *S. iniae* resisted phagocytosis at elevated temperatures via capsular polysaccharide (cps)-mediated mechanisms, revealing a novel immune escape strategy. These findings provide critical insights into the temperature-dependent immune evasion tactics employed by *S. iniae*.

## 2. Materials and Methods

### 2.1. Experimental Fish

Healthy experimental *A. latus* (20 ± 2 g mean weight) were obtained from the Nansha *A. latus* Hatchery (Guangzhou, China) and maintained at the Nansha-South China Agricultural University Fishery Research Institute, which is equipped with an automated LED lighting system (12L:12D photoperiod), a constant temperature control unit (maintained by carbon fiber heating element with PID feedback loops), and water recirculation filtration. Before experiments, the fish were kept in 260-liter glass tanks for two weeks. All animal experimental protocols were conducted according to the ethics guidelines of the Animal Research Ethics Committee of South China Agricultural University (Approval No. 2020B022).

### 2.2. Survival Assay

The wild-type *S. iniae* NS-1 strain was previously isolated under laboratory conditions from the brain of a dying *A. latus* (farmed in a pond) in Nansha district, Guangzhou, Guangdong Province, China. The median lethal dose (LD50) of *S. iniae* NS-1 was about 1.2 × 10^4^ CFU/g. *S. iniae* NS-1 was inoculated into a fresh brain heart infusion (BHI; BD Biosciences, Franklin Lakes, NJ, USA) culture medium at a ratio of 1:50 (*v*/*v*) and cultured in constant temperature incubator shakers at 33 °C and 23 °C, respectively. Bacteria incubated at each temperature were exclusively inoculated into fish groups maintained at the corresponding temperature. The bacteria were collected until the stationary growth phase. The survival assay was conducted as early publications with some modifications [26]. The trial utilized 180 healthy *A. latus*, randomly divided into six groups with standardized sample sizes: four infected groups (30 fish each, totaling 120 fish) and two control groups (30 fish each, totaling 60 fish), ensuring balanced experimental conditions for comparative analysis of temperature-dependent virulence effects. *A. latus* was infected by intraperitoneal injection with 100 μL of 1 × 10^7^ CFU/mL or 1 × 10^8^ CFU/mL *S. iniae* NS-1 (experimental), and an equal volume of sterile phosphate-buffered saline (PBS) was used as a control. Then, fish were maintained at 33 °C and 23 °C, respectively. After infection, the clinical signs and mortality of *A. latus* were recorded.

### 2.3. Proteomic Sequencing

*S. iniae* NS-1 was inoculated into fresh BHI culture medium at a ratio of 1:50 (*v/v)* and cultured at 23 °C and 33 °C. The bacterial pellets were collected and washed three times with sterile PBS, and proteins were extracted according to the manufacturer’s recommended protocol of the B-PER™ Enzymatic Bacterial Protein Extraction Kit (Thermo Fisher Scientific, Waltham, MA, USA), and extracted bacterial proteins were sent to Biotree (Biotree Biotech Co., Ltd., Shanghai, China) for proteome sequencing. The biological replicates for each group consisted of 3 cases. A vanquish neo-UPLC (Thermo Fisher Scientific, Waltham, MA, USA) connected to an Astral instrument (Thermo Fisher Scientific, Waltham, MA, USA) was used to separate and analyze the peptides. Mobile phase A was H_2_O/0.1% formic acid. Mobile phase B was 80% acetonitrile/0.1% formic acid. The samples were eluted using an 8 min gradient. An Orbitrap analyzer was used to perform data-independent acquisition (DIA) in both profile and positive modes, and the raw MS files were processed using DIA-NN 1.8.1 software. The correlation coefficient between each sample was calculated through the Pearson algorithm. The degree of correlation between two samples is expressed by the correlation coefficient r. The closer the absolute value of *r* is to 1, the stronger the correlation between the two samples. Proteins with a *p* < 0.05 as determined by Student’s *t*-test, fold change ≥1.2 or ≤0.83, were considered differentially expressed. Subcellular localization of differentially expressed proteins was performed using the website wolf psort. The cluster of orthologous groups (COG) database was used to annotate the functions of differentially expressed proteins.

### 2.4. Leukocyte Isolation

*A. latus* head kidney and spleen leukocytes were harvested using methods similar to those in early studies, with some modifications [27,28]. Briefly, *A. latus* were anesthetized with a concentration of 40 mg/L 3-aminobenzoic acid ethyl ester (Aladdin, Wuhan, China) [29]. To prevent blood from contaminating the spleen and head kidney, the heparinized syringe was used to draw blood from the caudal vein. Then, the intact head kidney and spleen were dissected with aseptic dissection tools and placed into a sterile Petri dish containing 5 mL RPMI-1640 (Gibco, Waltham, MA, USA) with 10% fetal bovine serum (FBS; Gibco, Waltham, MA, USA), 100 μg/mL streptomycin, and 100 U/mL penicillin G (Sigma Aldrich, Munich, Germany). Subsequently, the tissue was gently aspirated with a 1 mL syringe, and the resulting mixed suspension was filtered through a 70 μm cell strainer (Corning Incorporated, Corning, NY, USA) to obtain the single-cell suspension. The single cell suspension was slowly added onto a 51%/34% discontinuous Percoll (GE Healthcare, Chicago, IL, USA). After centrifugation at 500× *g* for 40 min and 4 °C, leukocytes were harvested from the 34% and 51% Percoll interface layer and washed three times with RPMI-1640. Cell quantity was determined by 0.4% trypan blue (Sigma Aldrich, Munich, German) and adjusted to a concentration of 1 × 10^6^ cells/mL.

### 2.5. S. iniae Stimulation and qPCR Analysis

Leukocytes at a concentration of 1 × 10^7^ cells/mL were resuspended in RPMI-1640 medium with 10% FBS and then plated into 96-well culture plates (100 μL/well). *S. iniae* NS-1 were added to the above cells at an MOI (multiplicity of infection) of 1:20 (cell: *S. iniae*), and then the mixture was incubated at 33 °C and 23 °C, respectively. The mixture time points of 0, 3, 6, 12, and 24 h was collected for flow cytometric and real-time quantitative PCR (qPCR) analysis. The partial cell pellets at the above times were added to Trizol lysis buffer (Vazyme, Nanjing, China), and total RNA was extracted as per the manufacturer’s instructions (Yeasen, Nanjing, China). NanoDrop 2000 (Thermo Fisher Scientific, Waltham, MA, USA) and 1.0% agarose gel electrophoresis (Biowest, Nogent-le-Rotrou, France) were used to detect RNA quantity and quality, and the cDNA synthesis super mix (Yeasen, Nanjing, China) was used to synthesize the cDNA. As shown in Table 1, the primers for this study were designed using NCBI primer-blast and then sent to Shenzhen BGI Genomics Service Co., Ltd., Shenzhen, China, for primer synthesis. The qPCR SYBR Green Master Mix (Yeasen, Nanjing, China) and the qPCR CFX96 Touch Real-time PCR (Bio-Rad, Hercules, CA, USA) were used to detect the transcriptional levels of apoptosis-related cytokine. The cycle threshold (CT) values for each sample were standardized based on the values for *A. latus* elongation factor 1α (ef1α) and *β*-actin. The results were then compared to the control group, and changes in gene expression were identified using the 2^−∆∆Ct^ method.

### 2.6. Apoptosis, ROS, and NO Levels

Cell apoptosis is often divided into early apoptosis (phosphatidylserine externalization to the cell surface) and late apoptosis (loss of cell membrane integrity). Annexin V can effectively bind to phosphatidylserine in both early and late apoptosis, indicating the overall apoptosis level of cells [30,31]. To detect total cell apoptosis level, the head kidney leukocytes at the time of 12 h were stained with 5 μL Annexin V-FITC (Beyotime, Shanghai, China) in 1× Annexin V Binding Buffer for 15 min at room temperature. Cellular NO and ROS during the *S. iniae* NS-1 infection at time points of 0, 3, 6, 12, and 24 h were detected using an NO assay kit (Beyotime, Shanghai, China) and ROS detection kit (Beyotime, Shanghai, China). The experimental procedures were performed following the manufacturer’s guidelines. Flow cytometry (BD Biosciences, Franklin Lakes, NJ, USA) was used to detect cell apoptosis, NO, and ROS.

### 2.7. Bacterial Labeling and Phagocytosis Assay

The *S. iniae* cps-deficient mutant (Δcps) was constructed and donated by Dr. Defeng Zhang from the Pearl River Fisheries Research Institute, Chinese Academy of Fishery Sciences. *S. iniae* NS-1 and Δcps were inoculated into fresh sterile BHI (BD Biosciences, Franklin Lakes, NJ, USA) and placed in a constant temperature incubator shaker at 23 °C and 33 °C. The bacteria cultured to the logarithmic phase were collected and inactivated with 0.4% formaldehyde for 12 h. After that, the bacteria were washed with sterile PBS. Fluorescein isothiocyanate (FITC; Sigma Aldrich, Munich, German) was added into the bacterial solution, incubated at 23 °C and 33 °C, and shaken at 160 rpm for 2 h. The bacteria were washed three times with sterile PBS to remove the unlabeled fluorescent dye, and then flow cytometry was performed to detect the fluorescence intensity of the bacteria.

The phagocytic activity of *A. latus* leukocytes at different temperatures was measured as reported in previous publications [32]. The isolated head kidney leukocytes, yellow-green microspheres (0.5 μm or 1.0 μm diameter; Polysciences, Warrington, PA, USA), and FITC-conjugated *S. iniae* (NS-1 or Δcps) were added to a 1.5 mL tube at a cell/bead-to-cell/*S. iniae* ratio of 1:20. The mixture was incubated at 23 °C and 33 °C with 2.5% CO_2_ for 2 h, respectively. To remove the non-uptake microspheres and *S. iniae*, the suspensions were subjected to centrifugation at 100× *g* for 10 min, three times, following a 3% BSA (Sigma Aldrich, St. Louis, MO, USA) solution in PBS with 4.5% D-glucose (Sigma Aldrich, St. Louis, MO, USA). The cell pellets were resuspended in sterile PBS for subsequent analysis via flow cytometry.

### 2.8. Statistical Analysis

Data handling, statistical analysis, and graphing were performed using GraphPad Prism 8.02 (GraphPad Software, Boston, MA, USA). The study followed a completely randomized design with experimental groups randomly assigned to ensure unbiased comparisons. Normality was verified via the Shapiro–Wilk test (*α* = 0.05). When the F-test indicated that the variances of the two groups were significantly different, statistical analyses were performed to compare the values obtained in each experimental group using the unpaired Student *t*-test. Multiple comparisons were performed using one-way analysis of variance, followed by Tukey’s multiple comparison test. The data were expressed as the mean ± standard deviation (SD) of three independent experiments, with significance defined as * (0.01 < *p* < 0.05), ** (*p* < 0.01), and *** (*p* < 0.001).

## 3. Results

### 3.1. Survival Rate

**Figure 1:**
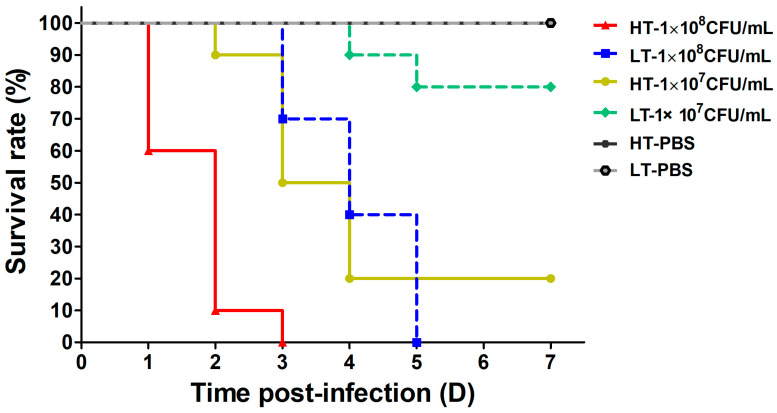
The survival rate of *A. latus* infected with *S. iniae* NS-1 at different temperatures. *A. latus* were infected by intraperitoneal injection with either different concentrations of *S. iniae* NS-1 (experimental) or an equal volume of sterile PBS buffer (control), and then fish were maintained in a high-temperature (HT, 33 °C) and low-temperature (LT, 23 °C) culture system, respectively. The cumulative survival of *A. latus* was assessed every day after infection until 7 days post-infection.

As shown in Figure 1, high-dose infection (1 × 10^8^ CFU/mL) resulted in 100% mortality of *A. latus* within three days at 33 °C, whereas at 23 °C, complete mortality was delayed until day 5. In the low-dose group (1 × 10^7^ CFU/mL), the cumulative survival rate at 33 °C was 20%, with mortality first observed at 2 days post-injection (dpi) and escalating rapidly during days 3–4. In contrast, at 23 °C, mortality remained at 10% until 4 dpi, reaching a total of 20% by 7 dpi. No mortality or clinical symptoms were observed in either control group. These results show that higher temperatures significantly accelerate mortality in *A. latus*, suggesting that temperature may be involved in regulating the virulence of *S. iniae* in *A. latus*.

### 3.2. S. iniae NS-1 Proteomic Sequencing

**Figure 2:**
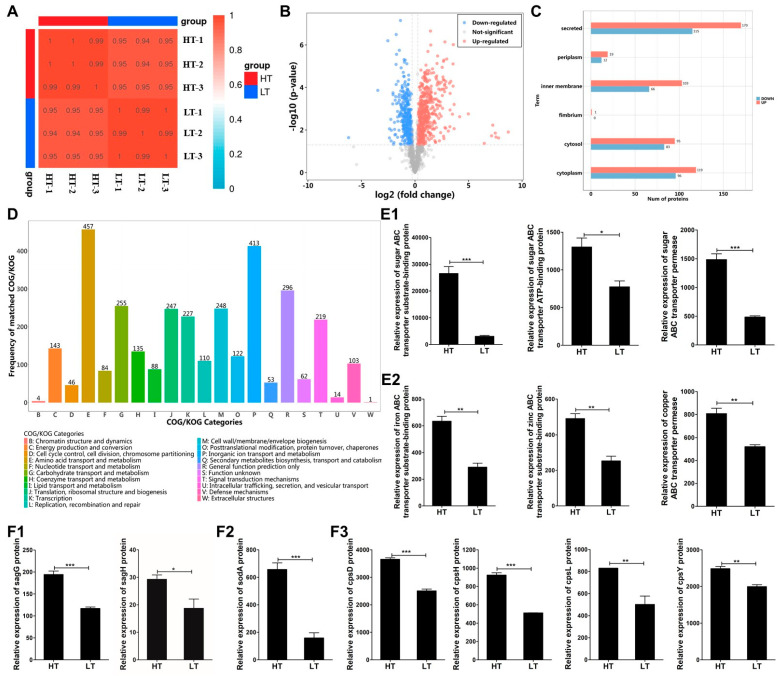
Proteomic analysis of *S. iniae* NS-1 at different temperatures. (**A**) Heatmap of correlation between samples. In this experiment, three biological replicates were set for each temperature, where HT-1, HT-2, and HT-3 represent three parallel samples at 33 °C, and LT-1, LT-2, and LT-3 represent three parallel samples at 23 °C. (**B**) Volcano plot of differentially expressed proteins. Red indicates upregulated proteins, blue indicates downregulated proteins, and gray indicates non-significantly differentially expressed proteins. (**C**) Histogram of subcellular localization analysis of differentially expressed proteins. Red represents upregulation and blue represents downregulation. (**D**) COG analysis histogram of differentially expressed proteins. Relative expression of sugar ABC transporters (**E1**), metal ion ABC transporters (**E2**), and virulence-associated proteins, including streptolysin S (SLS)-related proteins SagG and SagH (**F1**), antioxidant-related protein SodA (**F2**), and capsular polysaccharide synthesis-related proteins cpsD, cpsH, cpsL, and cpsY (**F3**). The data were expressed as the mean ± standard deviation (SD) of three independent experiments, with significance defined as * (0.01 < *p* < 0.05), ** (*p* < 0.01), and *** (*p* < 0.001).

The results of the correlation heatmap between samples showed that the correlation coefficients of parallel samples at the same temperature were above 0.99, and the correlation coefficients of parallel samples at different temperatures were above 0.94 (Figure 2A), indicating that there was a strong degree of correlation between the samples and could be used for subsequent analysis. The volcano plot showed that a total of 871 significantly differentially expressed proteins were screened, of which 504 were upregulations and 367 were downregulations (Figure 2B). The subcellular localization analysis histogram intuitively reflected that the differentially expressed proteins had strong mapping in secretory, endomembrane, and cytoplasm (Figure 2C). The result of COG analysis showed that the differentially expressed proteins were mainly enriched in inorganic ion transport and metabolism, carbohydrate transport and metabolism, and cell wall/membrane/envelope biosynthesis (Figure 2D). Among them, sugar ABC transporters (Figure 2(E1)) and metal ion transporters (Figure 2(E2)) were significantly upregulated at 33 °C. Virulence factors play a crucial role in bacterial immune escape; the expression of streptolysin S (SLS)-related proteins SagG and SagH (Figure 2(F1)), antioxidant-related protein SodA (Figure 2(F2)), and cps synthesis-related proteins cpsD, cpsH, cpsL, and cpsY (Figure 2(F3)) was significantly upregulated at 33 °C compared to 23 °C.

### 3.3. Leukocyte Kinetics After Stimulation

**Figure 3:**
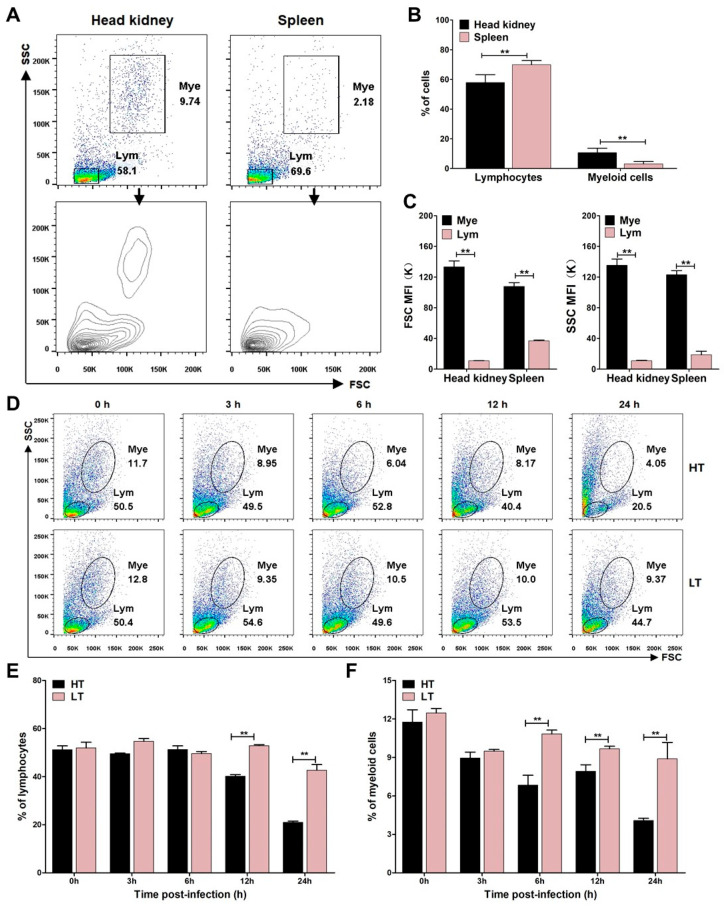
The dynamics of *A. latus* leukocyte populations during *S. iniae* NS-1 infection at different temperatures. (**A**) Morphological distribution of leukocyte subsets in *A. latus* head kidney and spleen, analyzed by flow cytometry. The leukocytes were classified into lymphocytes (Lym) and myeloid cells (Mye) based on scatter and contour plots. (**B**) Proportional comparison of lymphocytes and myeloid cells in head kidney versus spleen. (**C**) Mean fluorescence intensity (MFI) of forward scatter (FSC) and side scatter (SSC) for Mye and Lym populations in both tissues. (**D**) Representative flow cytometry scatter plots depicting Lym and Mye percentages in head kidney leukocytes after in vitro challenge with *S. iniae* NS-1 at high temperature (HT, 33 °C) and low temperature (LT, 23 °C). The percentage of lymphocytes (**E**) and myeloid cells (**F**) was summarized from the scatter plots. The error bars represent SD (*n* = 3). Significant difference is denoted by ** (*p* < 0.01).

In teleost fish, leukocyte populations can be classified into lymphocyte and myeloid cell populations [33,34,35]. In this study, leukocytes were successfully isolated from *A. latus* head kidney and spleen. Flow cytometry analysis revealed distinct clustering patterns: lymphocyte population was present in both tissues, whereas myeloid cell population was predominantly concentrated in the head kidney (Figure 3A,B). Additionally, myeloid cells exhibited markedly higher mean fluorescence intensity (MFI) values for both forward and side light scatter (FSC/SSC) than lymphocytes in both tissues (Figure 3C), indicating greater cellular complexity and size. These findings suggest that the head kidney serves as a primary reservoir for myeloid cells in *A. latus*, with these cells showing more pronounced aggregation than in the spleen. The lymphocyte population in teleost primarily consists of B cells, T cells, and NK-like cells, while myeloid cells include monocytes/macrophages and granulocytes [36]. Notably, teleost B cells, along with monocytes/macrophages and granulocytes, possess robust phagocytic and microbicidal capabilities [34]. Given these functional properties, head kidney leukocytes were selected for further investigation of immune evasion mechanisms during temperature-dependent *S. iniae* interactions. Dynamic analysis of leukocyte populations following *S. iniae* NS-1 stimulation at different temperatures revealed significant temporal changes (Figure 3D). Compared to the 23 °C group, the 33 °C group showed a substantial reduction in both lymphocyte (Figure 3E) and myeloid cell (Figure 3F) proportions within head kidney leukocytes at 12 h post-infection. There was no noticeable change in the ratio of lymphocytes and myeloid cells in the PBS group within 24 h (Figure S1). This temperature-dependent modulation of leukocyte populations highlights the potential impact of environmental factors on immune cell dynamics during *S. iniae* challenge.

### 3.4. Levels of Apoptosis

**Figure 4:**
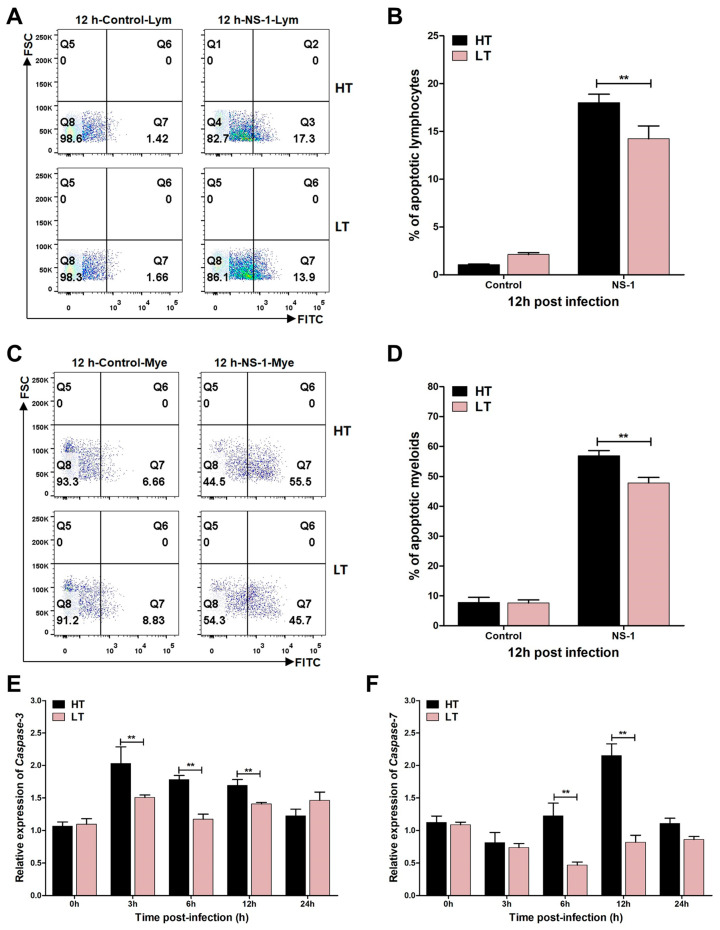
Effects of *S. iniae* NS-1 on apoptosis of head kidney lymphocytes and myeloid cells at different temperatures. (**A**,**C**) The scatter plots of flow cytometry showing the apoptosis of *A. latus* head kidney lymphocytes (**A**) and myeloid cells (**C**) after challenge with *S. iniae* NS-1 for 12 h at high temperature (HT, 33 °C) and low temperature (LT, 23 °C). (**B**,**D**) The percentage of apoptotic head kidney lymphocytes (**B**) and myeloid cells (**D**) was summarized from the fluorescence scatter plots. (**E***,***F**) The mRNA expression of the pro-apoptotic genes *caspase-3* (**E**) and (**F**) *caspase-7* was normalized to reference genes (*ef1α* and *β*-actin). The results were further compared with the control group to identify changes in gene expression by the 2^−ΔΔCt^ method. The error bars represent SD (*n* = 3). Significant difference is indicated by ** (*p* < 0.01).

Building on the observed dynamics of leukocyte populations post-infection, we quantitatively assessed apoptotic responses and pro-apoptotic gene expression profiles. The results demonstrated a temperature-dependent enhancement of *S. iniae* NS-1-induced apoptosis, with significantly elevated apoptosis rates in both lymphocytes (Figure 4A,B) and myeloid cells (Figure 4C,D) at 33 °C compared to 23 °C at 12 h post-stimulation. This thermal potentiation of apoptosis was further corroborated at the molecular level, showing marked upregulation of key executioner caspases with *caspase-3* (Figure 4E) and *caspase-7* (Figure 4F) mRNA expression levels being 1.2- and 2.6-fold higher, respectively, in the 33 °C group versus the 23 °C at 12 h post-stimulation.

### 3.5. Levels of ROS and NO

**Figure 5:**
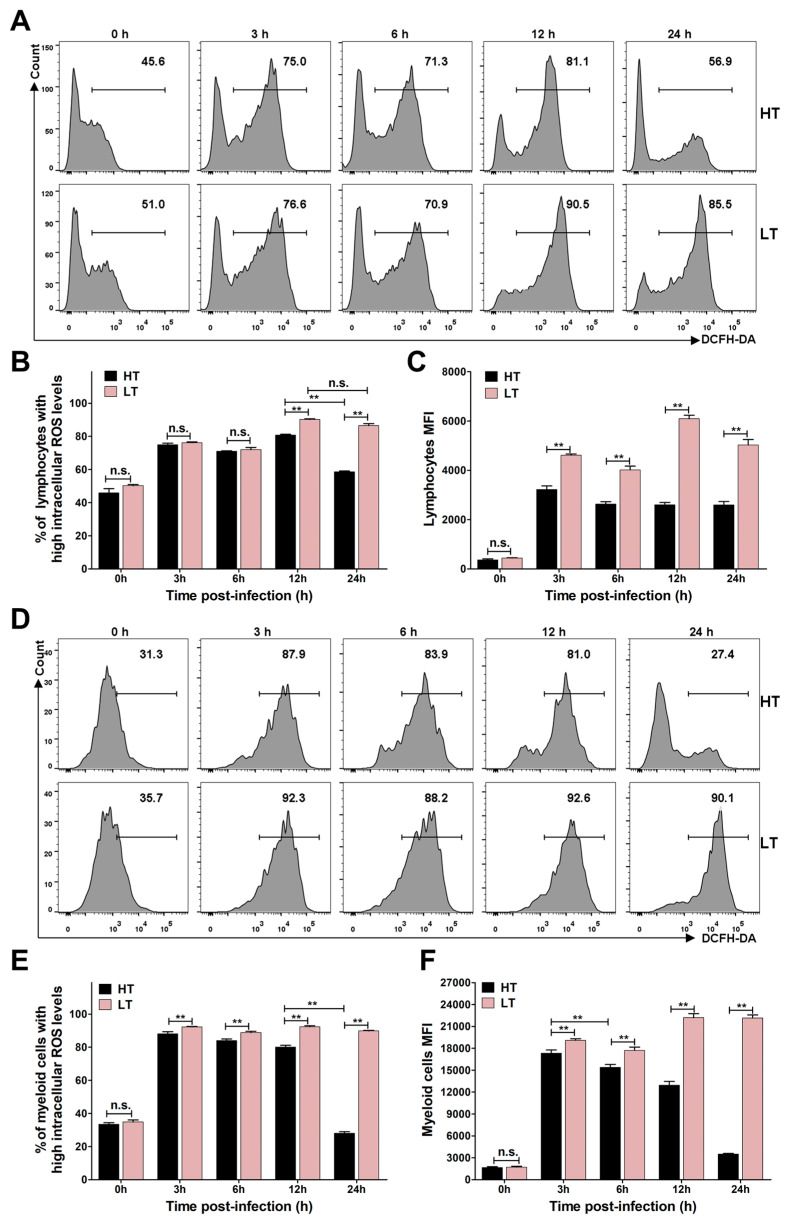
The intracellular ROS during *S. iniae* NS-1 infection at different temperatures. (**A**,**D**) The histogram of flow cytometry showing the intracellular ROS activity of *A. latus* head kidney lymphocytes (**A**) and myeloid cells (**D**) after stimulation with *S. iniae* NS-1 at HT (33 °C) and LT (23 °C). (**B**,**E**) The proportion of lymphocytes (**B**) and myeloid cells (**E**) with higher intracellular ROS were summarized from the fluorescence histogram. (**C**,**F**) Overall mean fluorescence intensity (MFI) of ROS in lymphocytes (**C**) and myeloid cells (**F**) was concluded from the fluorescence histogram. The error bars represent SD (*n* = 3). Significant difference is indicated by ** (*p* < 0.01); no significant difference is denoted by n.s. (*p* > 0.05).

**Figure 6:**
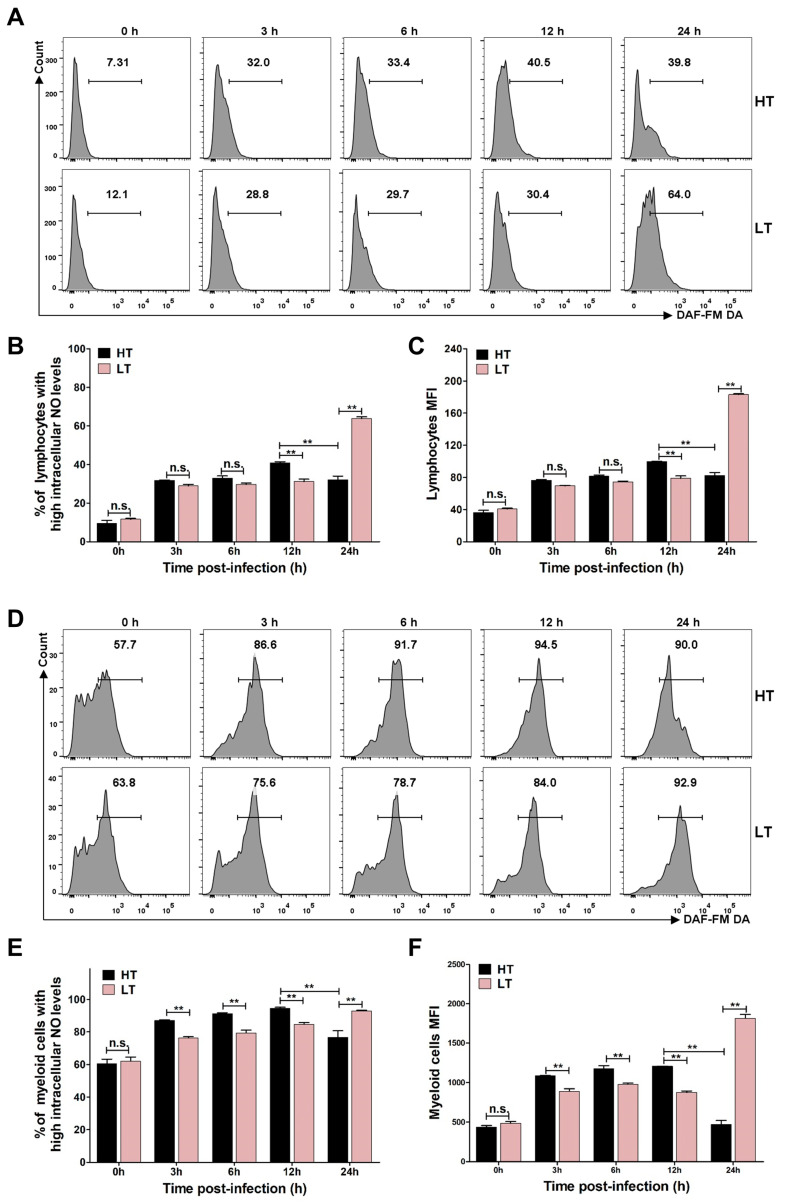
The intracellular NO during *S. iniae* NS-1 infection at different temperatures. (**A**,**D**) The histogram of flow cytometry showing the intracellular NO activity of *A. latus* head kidney lymphocytes (**A**) and myeloid cells (**D**) after stimulation with *S. iniae* NS-1 at high temperature (HT, 33 °C) and low temperature (LT, 23 °C). (**B**,**E**) The proportion of lymphocytes (**B**) and myeloid cells (**E**) with higher intracellular NO were summarized from the fluorescence histogram. (**C**,**F**) Overall mean fluorescence intensity (MFI) of NO in lymphocytes (**C**) and myeloid cells (**F**) was concluded from the fluorescence histogram. The error bars represent SD (*n* = 3). Significant difference is indicated by ** (*p* < 0.01); no significant difference is denoted by n.s. (*p* > 0.05).

Oxidative stress products, particularly ROS and NO, serve as potent bactericidal agents that play a critical role in host defense against invading pathogens. To evaluate the temperature-dependent effects of *S. iniae* NS-1 on oxidative stress responses, we quantified intracellular ROS (Figure 5) and NO (Figure 6) levels in *A. latus* leukocytes using flow cytometry. At 33 °C, *S. iniae* NS-1 significantly suppressed ROS production compared to 23 °C at 12 h post-stimulation, as evidenced by a reduced proportion of high-ROS lymphocytes (Figure 5B) and myeloid cells (Figure 5E), as well as decreased MFI of ROS in both lymphocytes (Figure 5C) and myeloid cells (Figure 5F). In contrast, NO production exhibited an inverse trend. At 33 °C, *S. iniae* NS-1 significantly increased the proportion with high intracellular NO and MFI of NO in both lymphocytes (Figure 6B,C) and myeloid cells (Figure 6E,F) compared to 23 °C at 12 h post-stimulation. In the PBS group, there was no noticeable change in the proportion of lymphocytes and myeloid cells containing high ROS and NO at 33 °C and 23 °C within 24 h (Figures S2 and S3). These divergent responses suggest that *S. iniae* NS-1 selectively disrupts ROS-mediated bactericidal activity at elevated temperature. This temperature-specific modulation of oxidative stress pathways may represent an adaptive immune evasion strategy, wherein the *S. iniae* targets the more potent ROS system to facilitate survival and proliferation in thermally stressed hosts.

### 3.6. Phagocytic Activity

**Figure 7:**
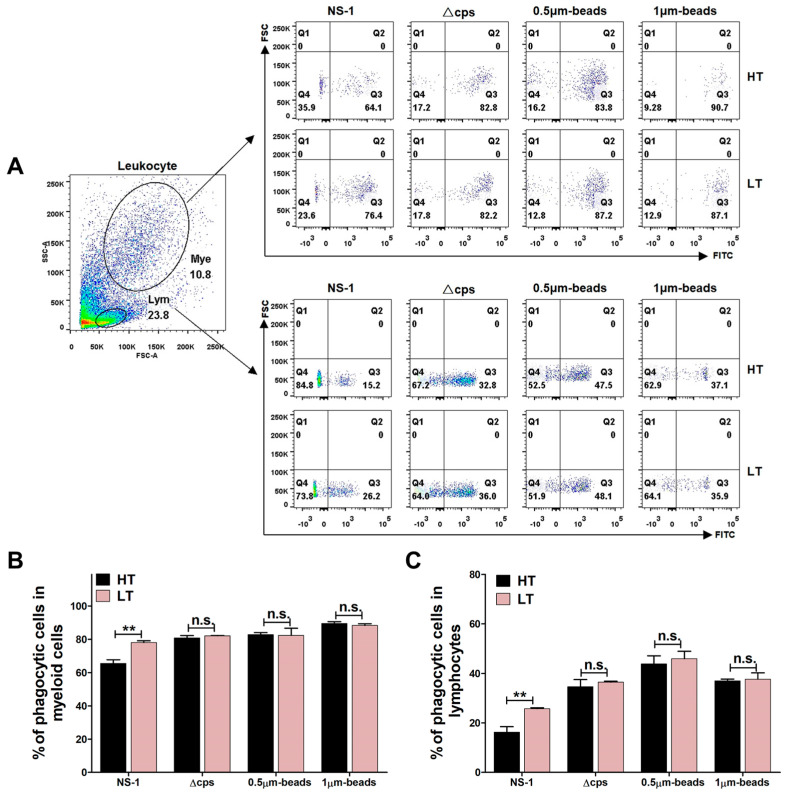
The phagocytic activity of lymphocytes and myeloid cells to *S. iniae* NS-1 at different temperatures. (**A**) Flow cytometry to evaluate phagocytosis of *S. iniae* (NS-1 or ∆cps) and fluorescent beads (0.5 μm or 1 μm) by myeloid cells (Mye, top) and lymphocytes (Lym, bottom) at high temperature (HT, 33 °C) and low temperature (LT, 23 °C). (**B**,**C**) The proportion of myeloid cells (**B**) and lymphocytes (**C**) that phagocytosed *S. iniae* and fluorescent beads. The results are representative of at least three independent experiments and are shown as mean ± SD. Significant difference is indicated by ** (*p* < 0.01); no significant difference is denoted by n.s. (*p* > 0.05).

To investigate the temperature-dependent resistance of *S. iniae* to phagocytosis, we analyzed the phagocytosis of FITC-labeled *S. iniae* strains (NS-1 and Δcps) and fluorescent beads (0.5 μm and 1.0 μm) by lymphocytes and myeloid cells using flow cytometry (Figure 7A). The results showed that the percentage of myeloid cells (Figure 7B) and lymphocytes (Figure 7C) phagocytosing *S. iniae* NS-1 was significantly reduced at 33 °C, while the percentage of phagocytic cells of *S. iniae* Δcps remained relatively constant at different temperatures. We also found that the phagocytic activity of lymphocytes and myeloid cells with 0.5 μm and 1.0 μm beads exhibited minimal change at 33 °C and 23 °C (Figure 7B,C), indicating that the temperature of 33 °C and 23 °C has no significant effect on cell phagocytosis. These findings suggest that *S. iniae* resisted phagocytosis at 33 °C through its cps.

## 4. Discussion

The strong correlation between elevated water temperatures and bacterial disease outbreaks in aquaculture is well documented, with pathogens such as *Streptococcus agalactiae* (*S. agalactiae*), *Edwardsiella*, and *Lactococcus* usually associated with rising water temperatures [37]. Specifically, *S. agalactiae* demonstrates low pathogenicity in tilapia at temperatures below 25 °C [38,39], whereas temperatures exceeding 27 °C significantly increase both the incidence of outbreaks and temperature-dependent mortality rates [40,41]. Our study extends this paradigm to *S. iniae* in *A. latus*, demonstrating that mortality rates are significantly higher at 33 °C compared to 23 °C, with survival rates inversely proportional to the bacterial injection dose (Figure 1). This aligns with prior observations in pompano (*Trachinotus ovatus*), where *S. iniae* induced severe mortality at 35 °C but not at 25 °C [16].

Temperature serves as a critical environmental regulator of virulence gene expression in fish pathogenic bacteria [37,42]. To elucidate the thermoregulated pathogenic mechanisms of *S. iniae*, we employed proteomic analysis to characterize temperature-dependent molecular responses. Comparative profiling revealed substantial transcriptional reprogramming at 33 °C, with 504 genes significantly upregulated compared to only 367 downregulated genes (Figure 2B). This predominant upregulation pattern aligns with previous transcriptomic observations at 35 °C, confirming a conserved thermal response in *S. iniae* [25]. The subcellular localization analysis revealed that differentially expressed proteins were predominantly localized to secretory pathways, endomembrane systems, and cytoplasm (Figure 2C), suggesting coordinated temperature-responsive adaptations in *S. iniae*. This regional distribution may reflect active secretion of virulence factors, cell wall/membrane remodeling, and metabolic regulation in response to thermal stress. COG analysis demonstrated significant enrichment of differentially expressed proteins in inorganic ion transport/metabolism and carbohydrate transport pathways (Figure 2D). Notably, multiple ABC transporter systems showed temperature-sensitive upregulation, including sugar ABC transporters (Figure 2(E1)) that facilitate host nutrient acquisition to support bacterial colonization and virulence as well as metal ion (iron, zinc, and copper) ABC transporters (Figure 2(E2)) that are essential for bacterial enzymatic activities and metabolic processes. These proteomic findings corroborate previous transcriptomic data showing elevated expression of ABC transport pathway components at 35 °C [25]. Virulence factors serve as critical mediators of bacterial immune evasion, with *S. iniae* employing a multifaceted strategy to subvert host defenses [43]. Using proteomics, we found a temperature-dependent virulence activation in *S. iniae* NS-1, with a significant upregulation of key pathogenic factors involved in invasion, resistance to killing, and phagocytosis at 33 °C (Figure 2(F1–F3)).

The maintenance of immune cell viability is crucial for effective pathogen recognition and the initiation of an immune response in the host [44]. Streptolysin S is a potent cytolysin produced by *S. iniae* that can induce the apoptosis and necrosis of neutrophils, lymphocytes, and erythrocytes [13,45]. Our study revealed that the expression of streptolysin S-related proteins SagG and SagH was significantly upregulated at 33 °C (Figure 2(F1)), suggesting that the cytolytic capacity of *S. iniae* NS-1 on immune cells was enhanced. To investigate the functional consequences of this temperature-dependent streptolysin S upregulation, we analyzed the temporal dynamics of head kidney leukocyte populations following *S. iniae* NS-1 infection within 24 h. Flow cytometry demonstrated a marked reduction in both lymphocyte and myeloid cell populations at 33 °C compared to 23 °C within 12 h post-infection (Figure 3E,F); this reduction was associated with the marked upregulation of the caspases-3/7 (Figure 4E,F) and enhanced apoptosis in both lymphocyte and myeloid lineages at 33 °C (Figure 4B,D). The depletion of myeloid cell and lymphocyte populations results in an impaired immune state in fish, explaining why fish mortality due to streptococcal disease is higher during high temperature in summer [46].

Host immune cells employ ROS and NO as key mediators of oxidative stress to eliminate invading pathogens [47]. During host–pathogen interactions, bacterial pathogens can use their antioxidant enzyme systems to interfere with the host’s oxidative stress response. For instance, the survival rate of the sodA deletion mutant of *Streptococcus suis* in macrophages was only half that of the wild-type strain [48]. Similarly, sodA has been demonstrated to facilitate *Aeromonas hydrophila* resistance to ROS-mediated killing in fish macrophages [49]. Our current investigation revealed a significant temperature-dependent upregulation of sodA expression in *S. iniae* NS-1 at 33 °C (Figure 2(F2)), suggesting an adaptive mechanism to bolster resistance against immune cell-mediated oxidative killing. Specifically, we observed that *S. iniae* infection at 33 °C significantly disrupted the release of ROS in lymphocytes and myeloid cells (Figure 5A,D), while having no impact on the release of NO (Figure 6A,D). The selective inhibition of ROS production, without altering NO pathways, aligns with the known function of sodA [49,50]. This enzyme specifically scavenges superoxide radicals (O_2_^−^)—a primary precursor of ROS—but does not interfere with nitric oxide synthase (NOS) activity or NO degradation. The differential response further suggests that *S. iniae* may exploit host antioxidant defenses to evade immune clearance, while leaving NO-mediated antimicrobial mechanisms intact.

Phagocytosis serves as a highly efficient host defense mechanism for eliminating invading pathogens [51]. Once “foreign invaders” break through the physical barriers of the skin or mucous membranes, phagocytes can rapidly migrate to the infection site, engulfing and neutralizing the pathogens [52]. Cps is a pathogenic weapon employed by many bacteria and plays a crucial role in protecting pathogens from the host’s innate defense. Previous studies have demonstrated that *S. iniae* cps mutant ΔcpsD exhibits enhanced sensitivity to phagocytic killing in both whole fish blood and fish macrophages, confirming that the cps can mediate resistance to cellular phagocytosis [53]. In the present study, we observed that 33 °C conditions induced a marked upregulation of key cps biosynthesis proteins, including cpsD, cpsH, cpsL, and cpsY (Figure 2(F3)), suggesting that *S. iniae* NS-1 cps synthesis is temperature-dependent. This finding aligns with prior reports in *Escherichia coli* [54,55], where specific cps-related genes also exhibit temperature-responsive expression patterns. To further investigate the functional role of cps in temperature-mediated anti-phagocytosis of *S. iniae*, we compared the phagocytic activity of *A. latus* lymphocytes and myeloid cells against the *S. iniae* NS-1 and Δcps at 23 °C and 33 °C. Notably, the phagocytic activity against Δcps remained unchanged across temperatures, while NS-1 showed significantly reduced uptake at 33 °C (Figure 7), indicating that cps mediates temperature-dependent phagocytosis resistance in *S. iniae*. Combining proteomic analysis and phagocytic function experimental data, this study preliminarily elucidated the molecular mechanism by which *S. iniae* resists clearance by fish phagocytes by temperature-dependent upregulation of the cps biosynthesis pathway, revealing a new temperature-sensitive immune escape strategy.

## 5. Conclusions

This study revealed a temperature-dependent virulence mechanism in the pathogenic mechanism of *S. iniae* NS-1 in the *A. latus* host. Proteomic analysis revealed significant upregulation of bacterial virulence determinants at 33 °C versus 23 °C, including apoptosis-inducing factors, oxidative stress resistance components, and phagocytosis evasion machinery. Host–pathogen interaction analysis established that *S. iniae* NS-1 not only upregulates the expression of *caspase-3/7* and induces apoptosis in immune cells but also selectively disrupts ROS production, not NO. Most significantly, we confirmed that *S. iniae* NS-1 resists phagocytosis by upregulating cps protein expression at 33 °C, revealing the temperature-sensitive immune escape strategy of *S. iniae* NS-1 mediated by cps. These results collectively elucidate a sophisticated temperature-responsive immune evasion strategy in *S. iniae*, advancing our understanding of thermal adaptation in aquatic bacterial pathogens.

## Figures and Tables

**Table 1:** Primers for qPCR used in this study.

Primers	Nucleotide Sequence (5′-3′)	Product Size (bp)	Amplification Efficiency	Sequence ID
*β*-actin-F	CGAGAGGGAAATCGTGCGTGACA	189	0.97	XM_037087864.1
*β*-actin-R	AGGAAGGAAGGCTGGAAGAGGGC			
ef1α-F	TCGGCGGTATTGGAACTGTC	210	0.98	XM_037092652.1
ef1α-R	CGACGTATCCACGACGGATT			
caspase3-F	CCCCGTAGAAGCTGACTTCC	213	1.02	XM_037083014
caspase3-R	GCATCAAAGCCCGGTGAATG			
caspase7-F	GACAGATTCAGGTCCGCCAA	162	0.96	XM_037081625.1
caspase7-R	TTGCAGAGTGCCTGGACAAA			

## Data Availability

The data supporting the reported results of the study can be provided from the corresponding author on reasonable request.

## Supplementary Materials

The following supporting information can be downloaded at https://www.mdpi.com/article/10.3390/biology14080986/s1, Figure S1: Flow cytometry dot plots showing the percentages of lymphocytes (Lym) and myeloid cells (Mye) in the head kidney leukocytes of the PBS group at high temperature (HT, 33 °C) and low temperature (LT, 23 °C); Figure S2: The intracellular ROS in the PBS group at different temperatures; Figure S3: The intracellular NO in the PBS group at different temperatures.
