# Supplementary material for: Proteomic and Functional Analysis Reveals Temperature-Driven Immune Evasion Strategies of Streptococcus iniae in Yellowfin Seabream (Acanthopagrus latus)
Source: Biology (Basel). 2025 Aug 2;14(8):986. doi: 10.3390/biology14080986 (PMC12383933; doi:10.3390/biology14080986)

Supplementary Materials

Figure S1. Flow cytometry dot plots showing the percentages of lymphocytes (Lym) and myeloid cells (Mye) in the head kidney leukocytes of the PBS group at high temperature (HT, 33℃) and low temperature (LT, 23℃).

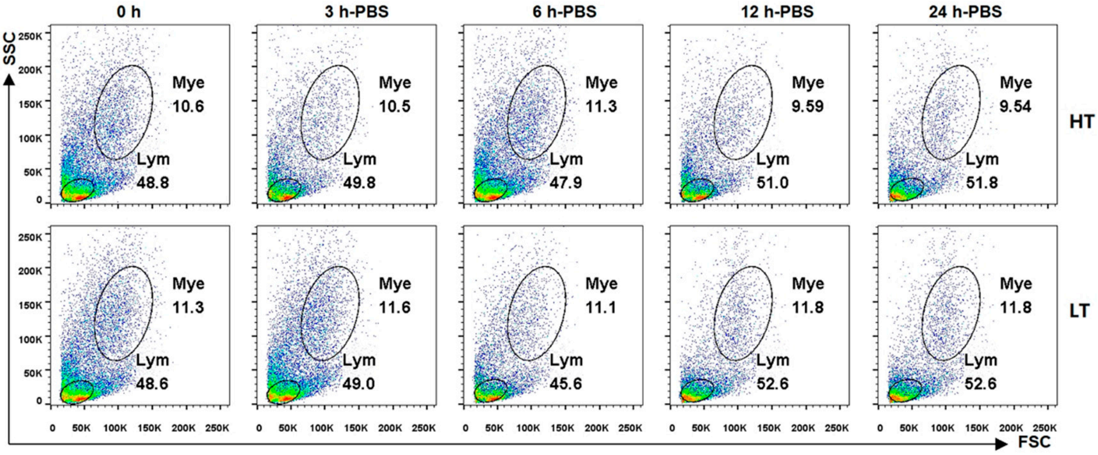

Figure S2. The intracellular ROS in the PBS group at different temperatures. The histogram of flow cytometry showing the intracellular ROS activity of *A. latus* head kidney lymphocytes **(A)** and myeloid cells **(B)** at high temperature (HT, 33℃) and low temperature (LT, 23℃).

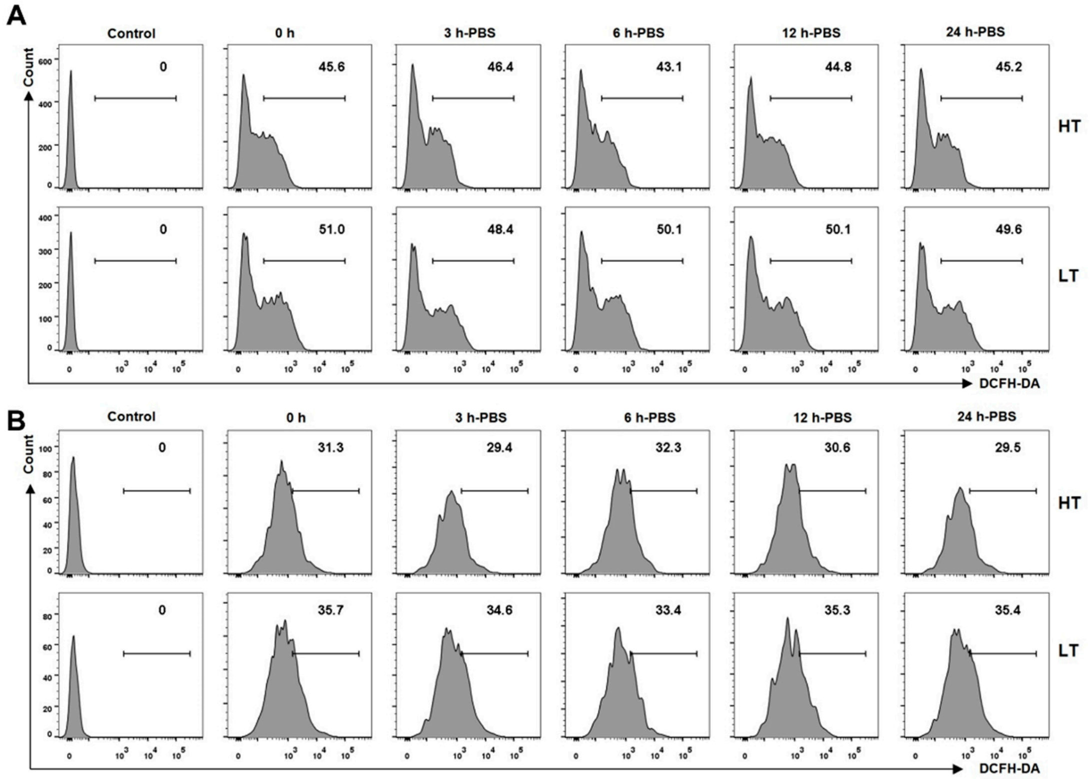

Figure S3. The intracellular NO in the PBS group at different temperatures. The histogram of flow cytometry showing the intracellular ROS activity of *A. latus* head kidney lymphocytes **(A)** and myeloid cells **(B)** at high temperature (HT, 33℃) and low temperature (LT, 23℃).

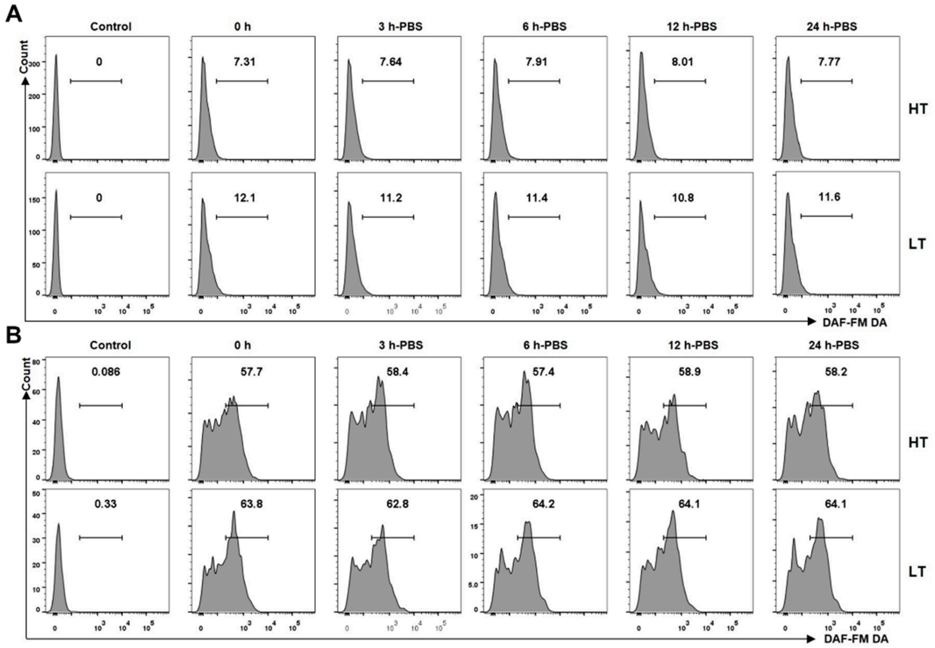

Supplement: Supplementary file 1 [file biology-14-00986-s001.zip › biology-3786592-supplementary.pdf]
